# A Single-Nucleotide Polymorphism of α_V_β_3_ Integrin Is Associated with the Andes Virus Infection Susceptibility

**DOI:** 10.3390/v11020169

**Published:** 2019-02-20

**Authors:** Constanza Martínez-Valdebenito, Jenniffer Angulo, Nicole Le Corre, Claudia Marco, Cecilia Vial, Juan Francisco Miquel, Jaime Cerda, Gregory Mertz, Pablo Vial, Marcelo Lopez-Lastra, Marcela Ferrés

**Affiliations:** 1 Departamento de Enfermedades Infecciosas e Inmunologia Pediatricas, División de Pediatría, Facultad de Medicina, Pontificia Universidad Católica de Chile, Santiago 8330024, Chile; cmartinezv@med.puc.cl (C.M.-V.); n.lecorre.p@gmail.com (N.L.C.); claumarco@gmail.com (C.M.); malopez@med.puc.cl (M.L.-L.); 2 Laboratorio de Virología Molecular, Instituto Milenio de Inmunología e Inmunoterapia (IMII), Santiago 8330024, Chile; jenniffer.at@gmail.com; 3 Facultad de Medicina, Center for Genetics and Genomics, Clínica Alemana Universidad del Desarrollo, Santiago 7650568, Chile; mcvial@udd.cl; 4 Departamento de Gastroenterologia, Escuela de Medicina, Pontificia Universidad Católica de Chile, Santiago 8330024, Chile; jfmiquel@med.puc.cl; 5 Facultad de Medicina Departamento de Salud Pública, Pontificia Universidad Católica de Chile, Santiago 8330024, Chile; jcerda@med.puc.cl; 6 University of New Mexico Health Sciences Center, Albuquerque, NM 87131, USA; gmertz@salud.unm.edu; 7 Departamento de Pediatria, Facultad de Medicina, Clínica Alemana Santiago, Universidad del Desarrollo, Santiago 7650568, Chile; pvial@udd.cl

**Keywords:** ANDV, SNP, PSI domain, L33P, rs5918, Hantavirus

## Abstract

The *Andes* *Orthohantavirus* (ANDV), which causes the hantavirus cardiopulmonary syndrome, enters cells via integrins, and a change from leucine to proline at residue 33 in the PSI domain (L33P), impairs ANDV recognition. We assessed the association between this human polymorphism and ANDV infection. We defined susceptible and protective genotypes as “TT” (coding leucine) and “CC” (coding proline), respectively. TT was present at a rate of 89.2% (66/74) among the first cohort of ANDV cases and at 60% (63/105) among exposed close-household contacts, who remained uninfected (*p* < 0.05). The protective genotype (CC) was absent in all 85 ANDV cases, in both cohorts, and was present at 11.4% of the exposed close-household contacts who remained uninfected. Logistic regression modeling for risk of infection had an OR of 6.2–12.6 (*p* < 0.05) in the presence of TT and well-known ANDV risk activities. Moreover, an OR of 7.3 was obtained when the TT condition was analyzed for two groups exposed to the same environmental risk. Host genetic background was found to have an important role in ANDV infection susceptibility, in the studied population.

## 1. Introduction

Hantaviruses, members of the Hantaviridae family, genus *Orthohantavirus*, are the etiological agents of two zoonotic diseases, known as hemorrhagic fever with renal syndrome (HFRS) and hantavirus cardiopulmonary syndrome (HCPS) [1,2]. Andes hantavirus (ANDV) is the sole etiological agent of HCPS in Chile and Southern Argentina, and its main reservoir is the long-tailed pygmy rice rat (*Oligoryzomys longicaudatus*) [3,4]. Through 14 July 2018, a total of 1141 cases of ANDV have been reported in Chile, with a lethality of 30% to 35% [5]. Transmission of ANDV to humans occurs mainly by exposure to aerosolized feces, urine, and saliva of infected rodents. However, ANDV person-to-person transmission has also been reported in Chile and Argentina [5,6,7].

After environmental or interpersonal virus exposure, the incubation period for ANDV infections has been estimated to be between 7 to 39 days, with an average of 18 days [6,8], while in the 2012 Yosemite outbreak, due to the Sin Nombre virus, the median incubation period was 30.5 days, with a range of 20–49 days [9]. Four stages characterize clinical presentation of HCPS, namely the prodromal, cardiopulmonary stage, diuresis, and convalescent phases. The prodromal phase is characterized by fever, headache, and myalgia. The cardiopulmonary phase presents with tachypnea and dry cough secondary to pulmonary edema that can quickly progress to respiratory failure, cardiogenic shock, and death, during this stage [1]. After several days, spontaneous diuresis occurs among survivors of the cardiopulmonary stage. The convalescent phase has been poorly characterized [8]. The first symptoms of the cardiopulmonary phase can progress rapidly to a severe disease with a need for mechanical ventilation (MV), the use of vasoactive drugs, and even the use of extracorporeal membrane oxygenation (ECMO). Strikingly, some patients exhibit a mild disease with only a minimal or total absence of oxygen supplement requirement [10,11].

Although risk factors for environmental and person-to-person transmission are well characterized, host factors that determine susceptibility to infection and disease severity are incompletely understood. In humans, pathogenic hantavirus, such as ANDV, replicate primarily in vascular endothelial cells. As such, differences in virus-cell affinity or the ability to attach to a known receptor might explain why a viral attachment and entry is successful. Endothelial cells infected with ANDV induce the production of the vascular endothelial growth factor (VEGF), followed by the downregulation of VE-cadherin, which leads to an increase in the microvascular permeability [12,13,14]. Several surface proteins and co-factors have been identified as mediators of virus entry and infection [12]. Viral interaction with β3 integrin induces the release of NET (neutrophil extracellular traps) [15]. Other examples of an infection-mediator is the DAF/CD55, in vitro assay that showed that this factor is critical for old hantavirus infections [16]. Recently, the PCDH1 protein was identified as an essential factor of entry and infection in pulmonary endothelial cells for single nucleotide polymorphism (SNP) and ANDV [17]. Additionally, in vivo and in vitro studies have identified integrin as one of the main cellular receptors used by hantaviruses [18,19,20]. The interaction between the envelope glycoproteins of ANDV and β3 integrin is mediated through the plexin–semaphorin–integrin (PSI) domain of the inactive integrin conformation [20].

It is noteworthy that the single nucleotide polymorphism (SNP rs5918) in human β3 integrin is a missense substitution (NP_000203.2:p.Leu59Pro) which equals the 33rd amino acid of the PSI domain (NP_000203.2:P.LEU59 Pro) and has been shown to reduce human β3 integrin–ANDV interaction [14]. This intriguing observation prompted us to design a study looking for a genetic association analysis, to address whether a link could be established between ANDV infection in Chilean patients and genetic variation in α_V_β_3_ integrin SNP rs5918. We predicted that if this was the case, individuals with SNP rs5918 leading to the (NP_000203.2:P.Leu59Pro) amino acidic substitution within the PSI domain would be less susceptible to an ANDV infection. To evaluate this possibility, samples from three groups of individuals were analyzed. The first group consisted of healthy individuals who were representative of the Chilean population [21]. The second group consisted of a case-close household contact population of individuals exposed to ANDV. This second group was further stratified to confirm ANDV-infected index cases and their close household contacts who remained uninfected during prospective follow-up. The third group consisted of household contacts who developed HCPS, during a prospective follow-up [6]. 

## 2. Materials and Methods

### 2.1. Study Population

#### Three Sets of Subjects Were Evaluated

Chilean Population (Group 1): For the first group, the general population, DNA samples from 477 non-related and ANDV-uninfected individuals were obtained from a well-characterized DNA library, harvested from a population considered to be representative of the Chilean population [21].

ANDV Cases and Close-Household Contacts Who Remained Uninfected (Group 2): In the second group, HCPS cases and close-household contacts were both exposed to ANDV. Briefly, 74 ANDV-infected individuals were confirmed through positive, specific immunoglobulin M serology or by positive ANDV reverse transcription-polymerase chain reaction (RT-PCR) [22,23]. A total of 105 close-household contacts were exposed to index cases and, in some cases, to common environmental risk factors, but remained uninfected during the five weeks of follow-up. These close-household individuals slept in the same bed or had close contact with an ANDV-infected patient for 30 days before and 7 days after the onset of HCPS symptoms. Both, the HCPS cases and close-household contacts were enrolled between 2008 and 2014. Demographic and epidemiological data were collected for cases and contacts through a previously validated questionnaire [6].

Household Contacts Who Developed HCPS During Prospective Follow-Up (Group 3): The third group included 11 subjects enrolled between 2002 and 2005 as healthy household contacts of ANDV cases who subsequently exhibited seroconversion and became ill during the five weeks of prospective follow-up [6]. DNA was available for 11 of the 14 household contacts who acquired ANDV infection.

### 2.2. Ethical Statement

Approval for the use of all samples and data and the research protocol design was obtained from the Ethical Review Board of the Facultad de Medicina, Pontificia Universidad Católica de Chile (Code 12-292 and 16-092). The participants or their legal representatives signed a written consent form, which was previously approved by the Ethical Review Board, at the time of enrollment.

### 2.3. DNA Extraction and Genotyping

Genomic DNA was extracted from cryopreserved blood samples, using the MagNApure compact system (Roche®, Mannheim, Germany), according to the manufacturer’s instructions. Genotyping of the rs5918 SNP was performed using a predesigned SNP assay with hydrolysis probes (ThermoFisher Scientific®, cat. n° 4351379). The amplification reaction was conducted using a Stratagene Mx3000P thermal cycler (Agilent Technologies, Santa Clara, CA, USA), and the assignment of alleles was performed automatically, by the MXPro QPCR software version 4.10 (Agilent Technologies), as described elsewhere [24], and manually reviewed by two independent investigators. To verify the correct assignment of alleles, control samples for each genotype (homozygote and heterozygote) were sequenced. Genotyping controls were added for each run, and all samples were run in duplicates.

In the logistic regression model, subjects with the TT genotype (homozygous for the major allele) were defined as “susceptible” to ANDV infection, and genotypes CT and CC (heterozygous and homozygous for minor alleles, respectively) were defined as “protective”.

### 2.4. Statistical Analysis

We used the software SPSS version 21 (SPSS, Inc., Chicago, IL, USA) for the descriptive analysis of each variable and the odds-ratio (OR) calculation (95% confidence interval). The frequency distribution for each variable was compared using Fisher’s exact test for contingency tables or the χ2 test, depending on the categorization of each variable. The χ2 test was used to verify any discrepancies of the SNP rs5918 distribution from the Hardy–Weinberg equilibrium. Significance was considered at *p* < 0.05.

A logistic regression model was employed to assess environmental or person-to-person risk factors for hantavirus infection, either in the presence or absence of the “susceptible” or “protective” genotype. In different multivariable models, for the genotype variable, we collapsed the “CC” genotype (codified to the proline or protective genotype) category with the “CT” genotype to avoid a zero value in the “CC” box for infected patients, for the regression modeling.

We calculated ORs using univariate modeling (OR crude) and three different strategies for the multivariate modeling. Briefly, the first, included all registered variables (OR1), the second (OR2) only included variables that were statistically significant in the univariate model (crude OR), and the third (OR3) only included variables described in the literature as risk factors involved in ANDV infections [6,10]. Additionally, we selected patients and household individuals who shared the same environmental exposure for evaluating the risk of ANDV infection for each genotype.

To compare the severity of ANDV-induced diseases and the SNP genotype, we assigned severe and mild categories, according to the patient’s clinical outcome. Mild disease was characterized as a febrile illness with nonspecific symptoms (e.g., headache, myalgia, chills, gastrointestinal symptoms) with no or minimal respiratory compromise. Severe cases were characterized for rapid and progressive impaired lung function, with mechanic ventilation and vasoactive drugs. Severe and mild were compared by the χ2 test, using the Graphad Prism version 7.04.

## 3. Results

### 3.1. Genotype Distribution in the General Population

**Figure 1:**
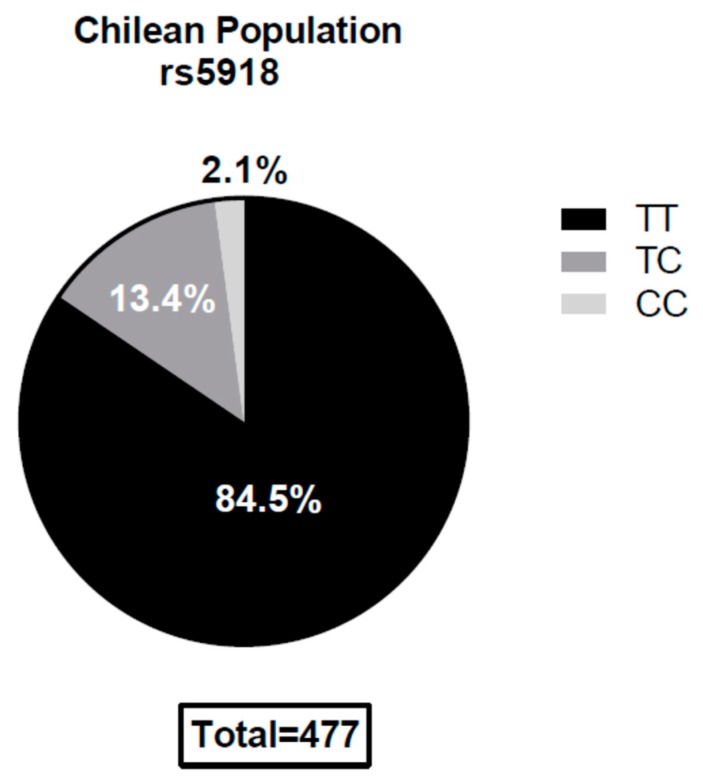
Single Nucleotide Polymorphism (SNP) rs5918 genotype distribution within the Chilean population. The TT genotype is the homozygous allele that codes for leucine at the 33rd position of the plexin–semaphorin–integrin (PSI) integrin domain. The CC genotype is the homozygous allele that codes for a proline at the same position, dramatically reducing *Andes Orthohantavirus* (ANDV) recognition in ex vivo models (14). The SNPs were in the Hardy–Weinberg equilibrium (*p* > 0.05).

Genomic DNA for 477 healthy individuals from a well-characterized DNA library considered to be representative of the Chilean population [21], was analyzed. The frequencies for the rs5918 TT, TC, and the CC genotypes were 84.5%, 13.4%, and 2.1%, respectively. The SNP rs5918 genotype was found to be in the Hardy–Weinberg equilibrium (χ2 tests *p* ≥ 0.1) (Figure 1).

### 3.2. Analysis of SNP rs5918 Distribution Among Study Group 2 (Cases and Close-Household Contacts) and Study Group 3 (11 Infected Close-Household Contacts)

**Figure 2:**
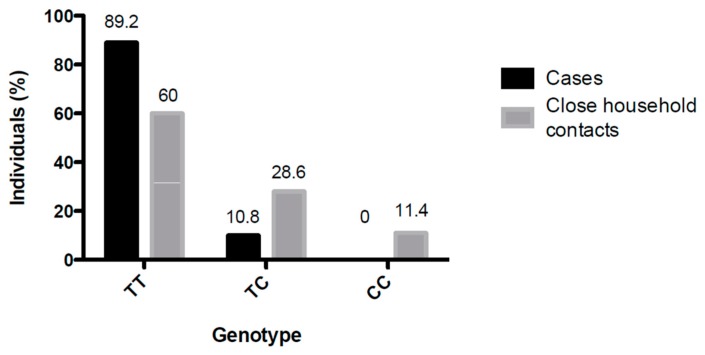
SNP rs5918 genotype distribution among cases and close-household contacts. The cases and household contacts were grouped according to the SNP rs5918 genotype. The total number of each population was defined as 100%, and the percentage of individuals according to each genotype was indicated (*p* > 0.05).

A higher distribution of the TT genotype was observed among the ANDV-infected subjects (89.2%) than among the close-household contacts (60%) (Figure 2). The protective CC genotype was absent from all ANDV-infected cases but present (11.4%) in exposed but not infected close-household contacts (*p* < 0.05). The TC genotype was found only in 10.8% of the ANDV-infected cases, but in 28.6% of the close-household contacts that remained uninfected (Table 1). Among the 11 household individuals who developed ANDV infection, five carried the TT genotype, 6 carried the CT genotype, and none carried the CC protective genotype.

Moreover, clear differences between the ANDV-infected patients and close-household individuals who remained uninfected were found for variables previously documented as risk factors for ANDV infection, such as cleaning or entering into abandoned places, handling wood, farm and forestry activities, and living in rural areas (Table 1).

### 3.3. ANDV Infection Risk Assessment and Risk Models for SNP rs5918 Genotype and Infection Among Cases and Close-Household Contacts

The risk of ANDV infection was assessed on the basis of the presence of the TT (susceptible) versus the CC/CT (defined as protective for the model) genotype and environmental variables. The crude OR for the existence of the TT genotype and ANDV-infection was 6.2 (CI: 2.7–14.1) (*p* < 0.05). When demographic and all exposure variables were added to the multivariable logistic model, the OR1 for the TT genotype increased to 19.7 (CI: 3–131). Finally, when we only included variables with a significant crude OR (model 2) and those that are well-recognized in the literature as relevant for ANDV infection (model 3), we obtained an OR2 and OR3 for the TT genotype of 12.6 (CI 2.9–55.3) for both (Table 2) *p* < 0.05.

### 3.4. ANDV Infection Risk Assessment Among Cases and Uninfected Close-Household Contacts Exposed to the Same Risk Activity

To rule out differences in exposure of cases and close-household contacts, we selected the two most frequent risk activities shared between ANDV-infected patients and close-household individuals who did not become infected, and assessed the OR of carrying the susceptible or protective genotype of SNP rs5918. When we related accessing an abandoned building, the susceptible genotype TT was present in 90.7% (39/43) of cases and in 57.1% (12/21) of close-household contacts. For wood handling, the TT genotype was present in 84.4% (38/45) of cases and 59.4% (19/32) of close-household contacts. The OR for ANDV infection in the presence of the TT genotype, for each activity, was 7.3 (1.9–28) and 3.7 (1.3–10.8), respectively (Table 3), *p* < 0.05.

### 3.5. Severity of ANDV-Induced Disease and SNP rs5918 Genotype Distribution

**Figure 3:**
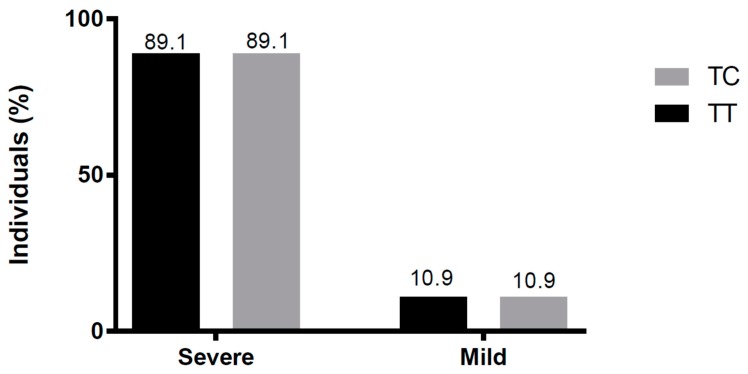
Genotype distribution among subjects with severe or mild diseases. Severe patients comprised four each with the TT or four CT genotype; TT and CT genotypes were present in 33 subjects with a mild disease.

We classified the cases as a mild or severe disease, according to the patient’s final clinical outcome. As shown in Figure 3, there were no differences in genotype distribution between severe and mild diseases (*p* > 0.99).

## 4. Discussion

Recent studies have linked the severity of ANDV infections to genetic factors. In this study, we sought to address whether the risk of infection may be associated with host variants, as an association between SNPs rs5918 and ANDV infection has been suggested by in vitro studies [14,20]. The ANDV-infected patients (74 patients) exhibited a frequency of 89.2% with regard to the susceptible genotype of the SNP rs5918, whereas the protective CC genotype was not found among these patients. Furthermore, none of the 11 close-household contacts who acquired an ANDV infection during a prospective follow-up, carried the CC genotype. In addition, the protective CC genotype was harbored by 11.4% of the exposed close-household individuals who did not become infected. These findings support the conclusion that the rs5918 TT genotype likely confers susceptibility to ANDV infection and that the rs5918 CC genotype seems to be protective.

Nevertheless, it is important to mention the marked differences in the frequency of the CC genotype, among the close-household contacts, compared to the Chilean population and other reports regarding rs5918, in which the frequency for this genotype was not more than 2% [25]. As mentioned above, the close-household contact population was exposed to the environmental risk factors of an ANDV infection, particularly person–person transmission. These individuals were the sexual partners of the patients, or parents or children of the patients, and therefore, blood related for the last two scenarios, which might explain the differences found in this particular cohort, compared to the Chilean population and previous reports on rs5918 [22].

Multiple in vitro studies have shown that the α_V_β_3_ integrin functions as the main receptor for entry of pathogenic hantaviruses, such as ANDV, and have suggested its role in the pathogenesis of the disease [14,18,19,20,26]. For example, Lui et al. showed that a polymorphism in the Human platelet alloantigen-3b (HPA-3b) allele (I843S) (integrin αIIbβ3) resulted in more severe clinical HFRS [27]. In addition, the NP_000203.2:P.Leu59Pro substitution in the PSI domain of β3, directs autoimmune responses through β3 integrins from blood containing a different HPA type, resulting in two autoimmune diseases involving vascular permeability and acute thrombocytopenia, similar to a hantavirus pathogenesis [28]. Thus, it is plausible that SNP rs5918 of β3 might also be associated with the ability of ANDV to infect.

The association between SNP rs5918 and hantavirus infection was recently studied in a Chinese population, and the authors failed to establish an association between SNP rs5918 and susceptibility to infection with Hantaan and Seoul viruses, both responsible for HRFS [29]

Nonetheless, due to differences between Old World and New World hantaviruses, in human illnesses, and in genetic differences between Chinese and Chilean populations, we evaluated the association between SNP rs5918 and ANDV infection [1,12]. Indeed, the Chilean population, which includes ancestral contributions from Europe, Native Americans, and a minor African component [30], differed sharply from the Asian cohort. In contrast to the results in the Chinese population, our results suggest that SNP rs5918 is associated with the ability of ANDV to infect Chilean subjects, suggesting that ethnic background should be accounted for, when establishing genetic studies. It should, however, be noted that genotype TT is prevalent among the general Chilean population, an observation that might bias our conclusions. Regardless, in support of our conclusion is the observation that the protective CC genotype was absent among the ANDV-infected patients and prevalent among the close-household contacts.

In vitro assays have established the critical role for ANDV infection of the leucine at site 33 of the β integrin PSI domain [14]. SNP rs5918 in the ITGB3 gene produces a Leu33Pro substitution; genotype TT results in a leucine at position 33, which is expected to facilitate the entry of the virus [12,14], whereas the CC genotype results in a proline that hinders binding of the ANDV glycoproteins to the integrin receptor [14]. Based on the observations in the present study, we evaluated the potential association between the risk of an ANDV infection and SNP rs5918, with the aim of understanding how the host’s genetic background impacts the distribution of an infectious disease.

As mentioned above, environmental risk factors for ANDV acquisition include, living in rural areas, working in forestry or agriculture, and recreational activities, such as camping or sleeping outdoors. Through logistic regression models, we weighed the impact of SNP rs5918 in the presence of hantavirus risk activities and assessed infection as the final outcome. The OR for the susceptible TT genotype was statistically significant in all models applied, highlighting the relevance of this β3 integrin polymorphism for ANDV infection. Although the CT and CC genotypes were considered to be protective for modeling purposes, data for individuals exhibiting SNP rs5918 heterozygosity are complex to interpret and should be regarded with caution. The rs5918 CT genotype is present in similar proportions among ANDV-infected patients, close-household contacts, and the general population. For the 11 prospective cases analyzed, it would be expected that TT was the most prevalent genotype; however, the CT distribution was higher, possibly due to the small sample size evaluated. It should be noted that as CC could better explain the role of SNP, as it has a lower frequency in the Chilean population and due to the large sample studied. Overall, which amino acid (Leucine or Proline) is encoded to be inserted at position 33 of the β3 protein among the heterozygous individuals for rs5918, remains unclear. Thus, without the direct amino acid sequence that is expressed in these cases, we cannot precisely determine whether the CT genotype represents a susceptible or protective condition. The high OR for SNP rs5918 in the three different regression models emphasizes its role in ANDV infection, in humans, and it would also be interesting to determine if this interaction between virus cells is the only characteristic responsible for the ability of ANDV to cause diseases in the Syrian hamster animal model or is one of the characteristic responsible for ANDV to be transmitted person-to-person [14].

One general characteristic of human infection is that not all individuals exposed to a pathogen become ill. To address this issue, different genetic markers have been associated with this broad range of susceptibility to infectious diseases. A good example is tuberculosis in the African population of Gana, Gambia, and Malawi, where an OR of 1.19 for developing tuberculosis illness has been found, for SNP rs4334126, which is located in a conserved region on 18q11.2, suggesting a possible regulatory effect on an unknown gene [31]. Here, we showed a significant difference in the distribution of SNP rs5918 NP_000203.2:P.Leu59Pro, with an OR of 7.3 and 3.7, for becoming infected, among patients and uninfected close-household contacts exposed to the same risk activities, respectively, suggesting a clear difference in susceptibility to ANDV infections.

In summary, using either the tested logistic regression models or the SNP rs5918 distribution in populations with the same risk activities, we were able to correlate the prevalence of SNP rs5918 to an ANDV SNP infection susceptibility. Nevertheless, other receptors and factors contribute to host susceptibility, globally. Our work highlights the relevance of the genetic background of the host to susceptibility to an infection and helps us understand why two equally exposed individuals have different infection outcomes.

## Figures and Tables

**Table 1:** SNP rs5918 genotypes and risk variable distribution among ANDV-infected patients and uninfected close-household individuals.

		ANDV Patients n/Total (%)	Close-Household Individuals n/Total (%)	
SNP	TT	66/74 (89.2)	63/105 (60)	*p* < 0.05
	TC	8/74 (10.8)	30/105 (28.6)	
	CC	0/74 (0.0)	12/105 (11.4)	
Age (mean)	Years old	36.1 (CI:32–40)	32.1 (CI:28.6–35.6)	
Sex	M	51/74 (68.9)	37/105 (35.2)	*p* < 0.05
Ethnicity	Hispanic	61/74 (82.4)	83/105 (79)	
	Native	6/74 (8.1)	7/105 (6.7)	
	Other	7/74 (9.5)	15/105 (14.3)	
Type of residence	Rural	38/74 (51.4)	40/105 (38.1)	
Work activities	High risk (forestry and agriculture)	26/74 (35.1)	8/105 (7.6)	*p* < 0.05
Risk activities	Visit rural areas	65/74 (87.8)	71/96 (74)	
	See rodents	31/73 (42.5)	17/97 (17.5)	*p* < 0.05
	See or touch rodent’s excrement	15/73 (20.5)	10/97 (10.3)	
	Handle gnawed food	12/73 (16.4)	3/97 (3.1)	*p* < 0.05
	Eat gnawed food	3/73 (4.1)	1/97 (1)	
	Rat extermination activities	10/73 (13.7)	3/97 (3.1)	
	Enter into abandoned shelter	43/73 (58.9)	21/97 (21.6)	*p* < 0.05
	Clean abandoned shelter	17/73 (23.3)	6/97 (6.2)	*p* < 0.05
	Clean up rodent’s feces	20/72 (27.8)	7/97 (7.2)	*p* < 0.05
	Forestry activities	20/73 (27.4)	12/97 (12.4)	
	Agricultural Activities	38/73 (52.1)	16/97 (16.5)	*p* < 0.05
	Handle wood	45/73 (61.6)	32/97 (33)	*p* < 0.05
	Walk into heavy forest	40/73 (54.8)	24/94 (25.5)	*p* < 0.05
	Collect wild fruits	17/73 (23.3)	15/97 (15.5)	*p* < 0.05
	Demolition activities	3/73 (4.1)	3/97 (3.1)	
	Camping in unauthorized areas	7/73 (9.6)	8/97 (8.2)	
	Sleep outdoors	6/74 (8.1)	3/97 (3.1)	

**Table 2:** Odds-ratios (ORs) for three logistic regression models linking genotypes and risk factors for ANDV acquisition. Data and numbers are shown in Table 1.

	OR Crude (CI)	OR^1^	OR^2^	OR^3^
SNP (TT)	6.2 (2.7–14.1) *	19.7 (3–131) *	12.6 (2.9–55.3) *	12.6 (2.9–54.5) *
Sex (M)	4.1 (2.2–7.7) *	1.3 (0.3–5.1)	1.5 (0.4–4.7)	1.3 (0.4–4.4)
Type of residence (R)	1.7 (0.9–3.1)	0.1 (0.02–0.8)	ND	ND
Work activities	8.8 (3.4–22.5) *	21 (2.5–178) *	7.9 (1.6–39.4)	8.1 (1.6–40.6)
Visit rural areas	2.5 (1.1–5.9) *	0.4 (0.05–3.6)	1.4 (0.4–5.1)	1 (0.2–5.6)
See rodents	3.5 (1.7–7) *	1 (0.2–5.4)	ND	ND
See or touch rodent’s excrement	2.2 (0.9–5.4)	0	ND	ND
Handle gnawed food	6.2 (1.7–22.7) *	8.3 (0.3–248)	1 (0–10.5)	ND
Eat gnawed food	4.1 (0.4–40.4)	1.9 (0.04–87)	ND	ND
Rat extermination activities	5 (1.3–18.8) *	4.4 (0.2–88)	1.2 (0–19.2)	
Get into abandoned shelter	5.2 (2.6–10.1) *	11 (2–60) *	3.3 (1–10.7)	4 (1.2–13)
Clean abandoned shelter	4.6 (1.7–12.4) *	0.6 (0.1–5.4)	0.8 (0.1–5.4)	0.8 (0.1–4.6)
Clean up rodent’s feces	5 (2–12.5) *	4.5 (0.3–68)	1.7 (0.2–17.1)	2.4 (0.4–14.7)
Forestry activities	2.7 (1.2–5.9) *	0.1 (0–0.6)	0.2 (0–1)	0.2 (0–1.2)
Agricultural activities	3.1 (1.5–6.4) *	0.2 (0–1.5)	0.9 (0.2–3.3)	0.8 (0.2–3.1)
Handle wood	3.2 (1.7–6.2) *	3.4 (0.7–16.3)	2.8 (0.8–9.8)	2.5 (0.7–9.4)
Walk into heavy forest	3.5 (1.8–6.8) *	38 (4–349) *	6.6 (1.8–23.4)	7.2 (2–25.3)
Collect wild fruits	1.7 (0.8–3.6)	3.7 (0.6–22)	ND	1.8 (0.5–6.8)
Demolition activities	1.3 (0.3–6.8)	0.3 (0–5)	ND	0.5 (0–8.5)
Camping in unauthorized areas	1.1 (0.4–3.4)	3.2 (0.3–33)	ND	1.2 (0.2–8.7)
Sleep outdoors	2.8 (0.7–11.4)	0.5 (0–6)	ND	ND

* *p* < 0.05; CI: the 95% confidence interval was used. OR^1^—all variables were included; OR^2^—only statistically significant variables were included; OR^3^—variables were selected according to the literature. ND = not determined.

**Table 3:** Distribution of SNP rs5918 genotypes among ANDV cases and uninfected close-household contacts exposed to the same risk activity.

Genotype	Access to Abandoned Places ^1^		Handle Wood ^2^	
	Cases n (%)	Close-household contacts n (%)	Cases n (%)	Close-household contacts n (%)
Susceptible (TT)	39 (90.7)	12 (57.1)	38 (84.4)	19 (59.4)
Protective (TC/CC)	4 (9.3)	9 (42.9)	7 (15.6)	13 (40.6)
**Total**	**43**	**21**	**45**	**32**

1 OR: 7.3 (IC: 1.9–28); 2 OR: 3.7 (IC: 1.3–10.8).

## References

[1] Schmaljohn C. (1997). Hantaviruses: A Global Disease Problem. Emerg. Infect. Dis..

[2] Knipe D.M., Howley P. (2013). Fields Virology (Knipe, Fields Virology)-2 Volume Set.

[3] Medina R.A., Torres-Perez F., Galeno H., Navarrete M., Vial P.A., Palma R.E., Ferres M., Cook J.A., Hjelle B. (2009). Ecology, Genetic Diversity, and Phylogeographic Structure of Andes Virus in Humans and Rodents in Chile. J. Virol..

[4] Toro J., Vega J.D., Khan A.S., Mills J.N., Padula P., Terry W., Yadón Z., Valderrama R., Ellis B.A., Pavletic C. (1998). An Outbreak of Hantavirus Pulmonary Syndrome, Chile, 1997. Emerg. Infect. Dis..

[5] Síndrome Cardiopulmonar por Hantavirus. http://epi.minsal.cl/hantavirus-materiales-relacionados/.

[6] Ferrés M., Vial P., Marco C., Yañez L., Godoy P., Castillo C., Hjelle B., Delgado I., Lee S., Mertz G.J. (2007). Prospective Evaluation of Household Contacts of Persons with Hantavirus Cardiopulmonary Syndrome in Chile. J. Infect. Dis..

[7] Martinez-Valdebenito C., Calvo M., Vial C., Mansilla R., Marco C., Palma R.E., Vial P.A., Valdivieso F., Mertz G., Ferrés M. (2014). Person-to-Person Household and Nosocomial Transmission of Andes Hantavirus, Southern Chile, 2011. Emerg. Infect. Dis..

[8] Vial P.A., Valdivieso F., Mertz G., Castillo C., Belmar E., Delgado I., Tapia M., Ferrés M. (2006). Incubation Period of Hantavirus Cardiopulmonary Syndrome. Emerg. Infect. Dis..

[9] Núñez J.J., Fritz C.L., Knust B., Buttke D., Enge B., Novak M.G., Kramer V., Osadebe L., Messenger S., Albariño C.G. (2014). Hantavirus Infections among Overnight Visitors to Yosemite National Park, California, USA, 2012. Emerg. Infect. Dis..

[10] Ferres M., Valdivieso F., Vial P.A. (2004). Manejo del Paciente Crítico con Sindróme Cardiopulmonar por Hantavirus.

[11] Ferrés M., Vial P. (1998). Hantavirus infection in children. Emerg. Infect. Dis..

[12] Vaheri A., Strandin T., Hepojoki J., Sironen T., Henttonen H., Mäkelä S., Mustonen J. (2013). Uncovering the mysteries of hantavirus infections. Nat. Rev. Microbiol..

[13] Borges E., Jan Y., Ruoslahti E. (2000). Platelet-derived Growth Factor Receptor β and Vascular Endothelial Growth Factor Receptor 2 Bind to the β _3_ Integrin through Its Extracellular Domain. J. Biol. Chem..

[14] Matthys V.S., Gorbunova E.E., Gavrilovskaya I.N., Mackow E.R. (2010). Andes Virus Recognition of Human and Syrian Hamster 3 Integrins Is Determined by an L33P Substitution in the PSI Domain. J. Virol..

[15] Raftery M.J., Lalwani P., Krautkrӓmer E., Peters T., Scharffetter-Kochanek K., Krüger R., Hofmann J., Seeger K., Krüger D.H., Schönrich G. (2014). β2 integrin mediates hantavirus-induced release of neutrophil extracellular traps. J. Exp. Med..

[16] Krautkrämer E., Zeier M. (2008). Hantavirus causing hemorrhagic fever with renal syndrome enters from the apical surface and requires decay-accelerating factor (DAF/CD55). J. Virol..

[17] Jangra R.K., Herbert A.S., Li R., Jae L.T., Kleinfelter L.M., Slough M.M., Barker S.L., Guardado-Calvo P., Román-Sosa G., Dieterle M.E. (2018). Protocadherin-1 is essential for cell entry by New World hantaviruses. Nature.

[18] Gavrilovskaya I.N., Brown E.J., Ginsberg M.H., Mackow E.R. (1999). Cellular Entry of Hantaviruses Which Cause Hemorrhagic Fever with Renal Syndrome Is Mediated by β3 Integrins. J. Virol..

[19] Gavrilovskaya I.N., Gorbunova E.E., Mackow N.A., Mackow E.R. (2008). Hantaviruses Direct Endothelial Cell Permeability by Sensitizing Cells to the Vascular Permeability Factor VEGF, while Angiopoietin 1 and Sphingosine 1-Phosphate Inhibit Hantavirus-Directed Permeability. J. Virol..

[20] Raymond T., Gorbunova E., Gavrilovskaya I.N., Mackow E.R. (2005). Pathogenic hantaviruses bind plexin-semaphorin-integrin domains present at the apex of inactive, bent v 3 integrin conformers. Proc. Natl. Acad. Sci. USA.

[21] Miquel J.F., Covarrubias C., Villaroel L., Mingrone G., Greco A.V., Puglielli L., Carvallo P., Marshall G., Pino G.D., Nervi F. (1998). Genetic epidemiology of cholesterol cholelithiasis among Chilean Hispanics, Amerindians, and Maoris. Gastroenterology.

[22] Valle M.O.D., Edelstein A., Segura E.L., Rossi C.M., Colavecchia S.B., Rabinovich R.D., MartíNez P.V., Miguel S.D.L., Padula P.J. (2000). Development and evaluation of a solid-phase enzyme immunoassay based on Andes hantavirus recombinant nucleoprotein. J. Med. Microbiol..

[23] Vial C., Martinez-Valdebenito C., Rios S., Martinez J., Vial P.A., Ferres M., Rivera J.C., Perez R., Valdivieso F. (2016). Molecular method for the detection of Andes hantavirus infection: validation for clinical diagnostics. Diagn. Microbiol. Infect. Dis..

[24] Angulo J., Pino K., Pavez C., Biel F., Labbé P., Miquel J.F., Soza A., López-Lastra M. (2013). Genetic variations in host IL28B links to the detection of peripheral blood mononuclear cells-associated hepatitis C virus RNA in chronically infected patients. J. Viral Hepat..

[25] Reference SNP (refSNP) Cluster Report: rs5918** with Pathogenic Allele**. https://www.ncbi.nlm.nih.gov/projects/SNP/snp_ref.cgi?rs=rs5918.

[26] Gavrilovskaya I.N., Peresleni T., Geimonen E., Mackow E.R. (2002). Pathogenic hantaviruses selectively inhibit β3 integrin directed endothelial cell migration. Arch. Virol..

[27] Liu Z., Gao M., Han Q., Fang J., Zhao Q., Zhang N. (2008). Intensity of Platelet β3 Integrin in Patients with Hemorrhagic Fever with Renal Syndrome and Its Correlation with Disease Severity. Viral Immunol..

[28] Liu Z., Gao M., Han Q., Lou S., Fang J. (2009). Platelet glycoprotein IIb/IIIa (HPA-1 and HPA-3) polymorphisms in patients with hemorrhagic fever with renal syndrome. Hum. Immunol..

[29] Chen X.-P., Xiong H.-R., Zhu N., Chen Q.-Z., Wang H., Zhong C.-J., Wang M.-R., Lu S., Luo F., Hou W. (2017). Lack of association between integrin α_V_β_3_ gene polymorphisms and hemorrhagic fever with renal syndrome in Han Chinese from Hubei, China. Virol. Sin..

[30] Eyheramendy S., Martinez F.I., Manevy F., Vial C., Repetto G.M. (2015). Genetic structure characterization of Chileans reflects historical immigration patterns. Nat. Commun..

[31] Chapman S.J., Hill A.V.S. (2012). Human genetic susceptibility to infectious disease. Nat. Rev. Genet..

